# Mathematical modeling of positron emission tomography (PET) data to assess radiofluoride transport in living plants following petiolar administration

**DOI:** 10.1186/s13007-015-0061-y

**Published:** 2015-03-13

**Authors:** Alexander K Converse, Elizabeth O Ahlers, Tom W Bryan, Jackson D Hetue, Katherine A Lake, Paul A Ellison, Jonathan W Engle, Todd E Barnhart, Robert J Nickles, Paul H Williams, Onofre T DeJesus

**Affiliations:** T123 Waisman Center, University of Wisconsin-Madison, Madison, WI 53705 USA; Department of Plant Pathology, University of Wisconsin-Madison, Madison, WI 53705 USA; Department of Medical Physics, University of Wisconsin-Madison, Madison, WI 53705 USA

**Keywords:** Ion transport, Positron emission tomography, Radiotracer, Kinetic modeling, Brassica oleracea

## Abstract

**Background:**

Ion transport is a fundamental physiological process that can be studied non-invasively in living plants with radiotracer imaging methods. Fluoride is a known phytotoxic pollutant and understanding its transport in plants after leaf absorption is of interest to those in agricultural areas near industrial sources of airborne fluoride. Here we report the novel use of a commercial, high-resolution, animal positron emission tomography (PET) scanner to trace a bolus of [^18^F]fluoride administered via bisected petioles of *Brassica oleracea*, an established model species, to simulate whole plant uptake of atmospheric fluoride. This methodology allows for the first time mathematical compartmental modeling of fluoride transport in the living plant. Radiotracer kinetics in the stem were described with a single-parameter free- and trapped-compartment model and mean arrival times at different stem positions were calculated from the free-compartment time-activity curves.

**Results:**

After initiation of administration at the bisected leaf stalk, [^18^F] radioactivity climbed for approximately 10 minutes followed by rapid washout from the stem and equilibration within leaves. Kinetic modeling of transport in the stem yielded a trapping rate of 1.5 +/− 0.3%/min (mean +/− s.d., n = 3), velocity of 2.2 +/− 1.1 cm/min, and trapping fraction of 0.8 +/− 0.5%/cm.

**Conclusion:**

Quantitative assessment of physiologically meaningful transport parameters of fluoride in living plants is possible using standard positron emission tomography in combination with petiolar radiotracer administration. Movement of free fluoride was observed to be consistent with bulk flow in xylem, namely a rapid and linear change in position with respect to time. Trapping, likely in the apoplast, was observed. Future applications of the methods described here include studies of transport of other ions and molecules of interest in plant physiology.

**Electronic supplementary material:**

The online version of this article (doi:10.1186/s13007-015-0061-y) contains supplementary material, which is available to authorized users.

## Background

Ion transport is a fundamental process in plant physiology [1-3]. Radiotracer methods have been useful for studying transport phenomena because of their inherent sensitivity and quantitative accuracy [4]. Positron emission tomography (PET) is a non-invasive radiotracer imaging technique widely used in the clinic to diagnose diseases and monitor their progression [5]. PET imaging provides tracer kinetic data on the uptake, distribution, retention and clearance of positron-emitting compounds administered to living organisms. These tracer kinetic data can then be modeled to obtain insights into trapping mechanisms from which information on metabolism [5], receptor density [6], and other *in vivo* molecular interactions can be obtained.

In this study PET imaging was utilized to model fluoride ion transport in plants following absorption through the leaves by administering radiofluoride via a cut petiole. Fluoride uptake and distribution in the whole living plant was then non-invasively monitored in real-time by PET. Time-activity curves (TACs) in selected regions of interest (ROIs) in the plant were obtained and mathematically modeled to assess fluoride transport rates and trapping probabilties.

Fluoride is a trace element ubiquitous in the environment. Fluoride has beneficial effects in human health such as in preservation of teeth, but excessive fluoride leads to toxic effects such as fluorosis of bones and teeth in humans and animals as well as browning of leaves in plants [7] (p.2). Environmental fluoride comes from both natural sources, e.g. volcanic emissions, and industrial sources, e.g. aluminum smelting [7] (pp. 6–18). Inorganic fluoride can be taken up by plants from soil through the root system and from air through the stomata of leaves [8]. Fluoride uptake via roots is mitigated by its low solubility in soil. While the highest concentration of fluoride in plants is found in the roots and diminishes with distance from the roots, 95% of the fluoride present in the roots can be readily washed away with water [7] (p. 33). Plant uptake of fluoride from air is more significant than uptake from soil [9]. Fluoride concentration in the leaves can range from < 1 mg/kg to several thousand mg/kg [7] (p. 32). Reviews of mechanisms of fluoride uptake through the leaves from air and their effects on plant metabolism and growth can be found in [7] (pp. 36–46), [10], and [8].

The kinetics of uptake and transport of [^18^F]fluoride in plants determined by positron emission imaging was first reported by McKay et al. [11] in a study utilizing a custom-made imaging system comprised of two planar detectors designed to detect coincident positron annihilation events. Later work by other investigators employed a more advanced positron-emitting tracer imaging system (PETIS) to follow plant uptake and transport of [^18^F]fluoride to study radiation effects [12] and drought effects [13]. In the present study, we used a commercially available, small-animal, high-resolution 32-ring PET scanner, which we had found to be well-suited for plant imaging [14,15].

In contrast to previous studies that utilized cut stems that were immersed in aqueous [^18^F]fluoride solution [11-13,16], the present study involved application of microliter amounts of aqueous [^18^F]fluoride solution into the freshly-cut petiole of our plant model, rapid cycling *Brassica oleracea* [17]. This technique allowed for the behavior of a bolus of fluoride traveling up the stem of the living plant to be characterized using kinetic modeling, which permitted estimation of trapping probability and mean position as a function of time. Based on previous studies, we hypothesized *a priori* that the tracer would appear to move at a uniform velocity along the stem [11,18].

## Results

**Figure 1:**
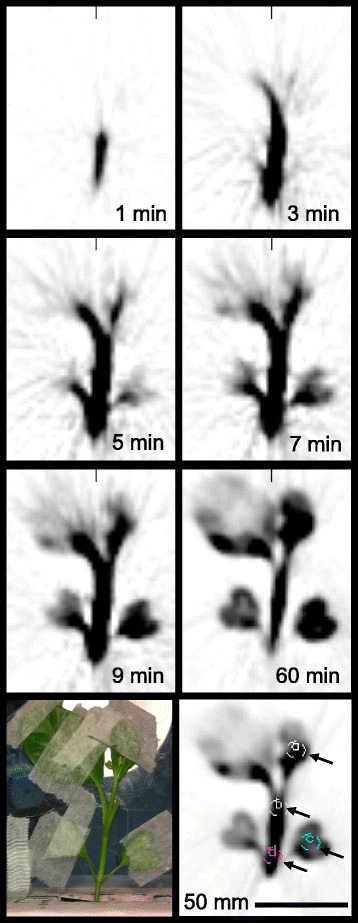
**[** ^**18**^ **F]fluoride uptake.** PET images shown in a 1.2 mm thick transverse scanner plane. Times shown relative to start of petiolar administration. bottom row: photograph of plant and four regions of interest (arrows) delineated on 0 to 60 minute image used for time-activity curves shown in Figure 2 (a = leaf, b = stem - high, c = cotyledon, d = stem - low).

**Figure 2:**
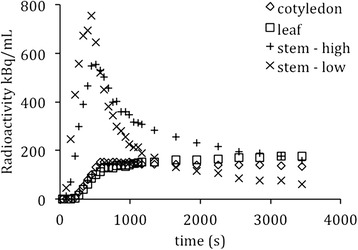
**Radioactivity in stem and leaves.** Time-activity curves corresponding to regions of interest shown in Figure 1. Radioactivity is seen moving as a bolus through the stem and accumulating in the leaves.

**Figure 3:**
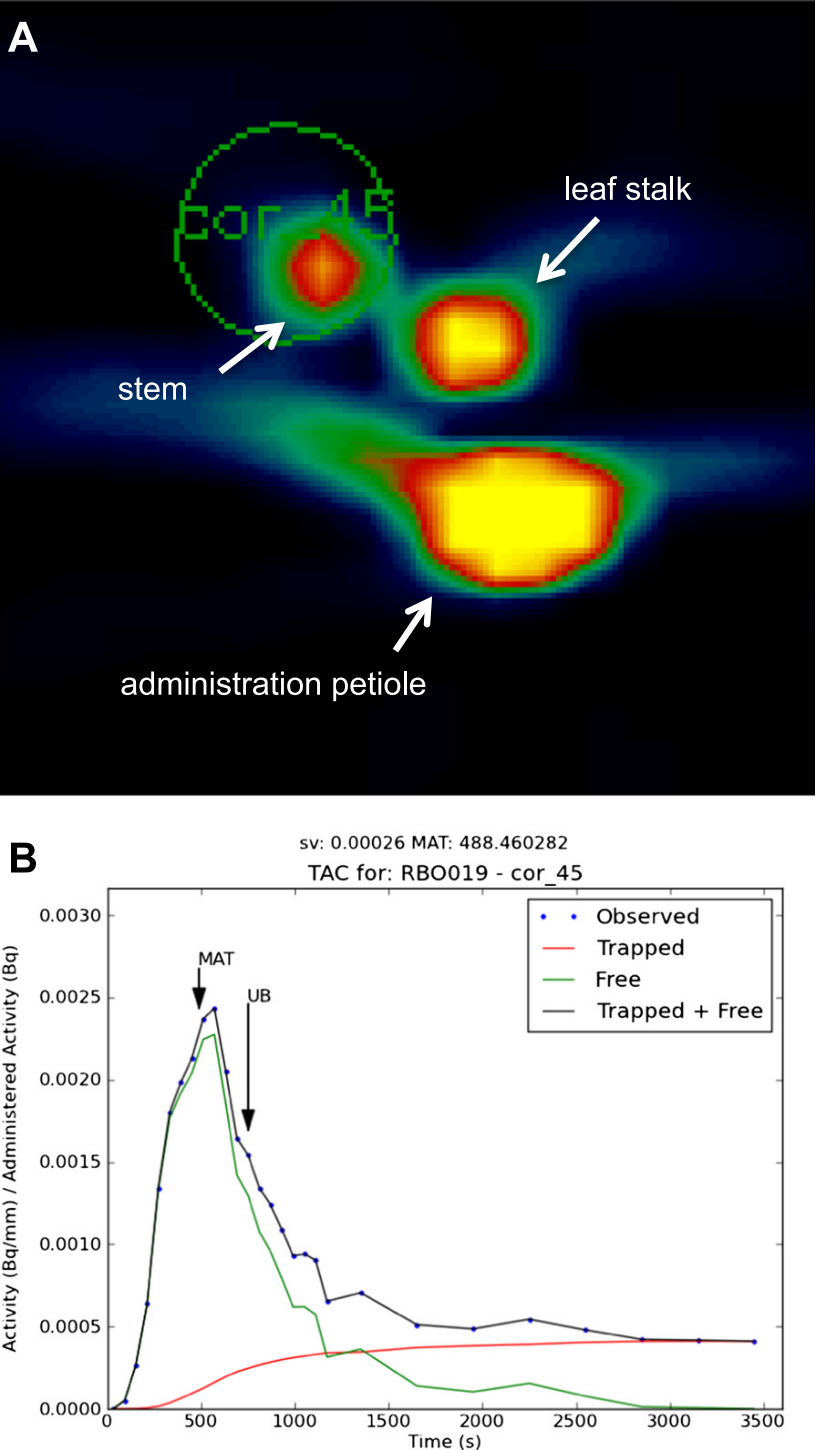
**Modeling of observed radioactivity in stem. (A)** 1.9 mm thick horizontal cross section of plant shown in Figure 1 at upper stem. **(B)** Time-activity curves showing observed activity in stem (blue), modeled free activity (green), modeled trapped activity (red), total modeled activity (black). MAT: mean arrival time, UB: upper bound of integral to calculate MAT.

**Figure 4:**
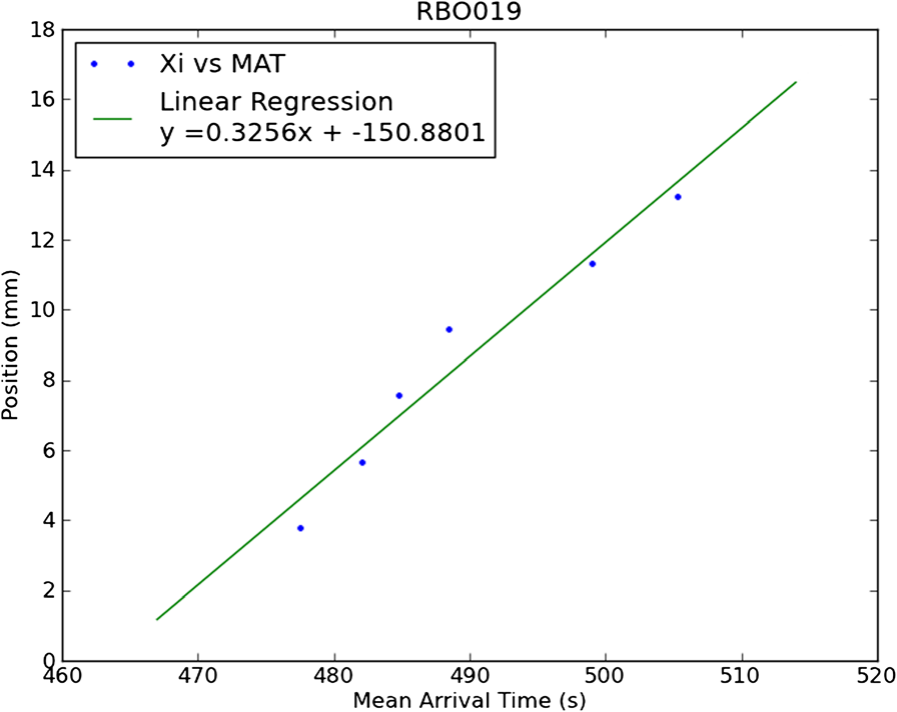
**Speed of bolus traveling up stem.** Plot of mean arrival time (s) at various positions along the stem (mm) with respect to the node of the bisected petiole used for administration. The slope gives the speed (mm/s) at which the bolus travels.

Three plants (*Brassica oleracea,* age 22.0 +/− 3.5 days), were imaged by PET for one hour starting with petiolar administration of [^18^F]fluoride (7.2 +/− 3.6 MBq) in 14 +/− 4 microliters of reverse osmosis water. PET images revealed uptake throughout the plant within ten minutes (Figure 1). Concentration over time is shown in Additional file 1: Movie 1, and the final distribution is shown in Additional file 2: Movie 2. As seen in Figure 2, time-activity curves for stems and leaves were consistent with bolus transport, and leaves exhibited greater relative trapping of radioactivity. A tracer kinetic model was implemented that describes the time courses of free and trapped [^18^F]fluoride. This model was used to determine a single parameter, the trapping probability per unit time, sv (1/s), which is the product of the trapping probability per unit length, s (1/mm), and the local velocity, v (mm/s), at a given position along the stem. Using this model, the time-activity curve for the free fluoride was inferred at various positions along the stem of the plants, and this was used to calculate the mean arrival time of the free fluoride at each of these positions (Figure 3). The velocity of transport up the stem, V, was determined based on the mean arrival time of the free fluoride at various positions along the stem (Figure 4). Averaging over results for the three plants, the observed trapping probability per unit time was sv = 0.000253 +/− 0.000053 1/s, and the mean velocity was 0.368 +/− 0.180 mm/s. Trapping probabilities and velocities for each of the three plants are detailed in Table 1.

**Table 1:** **Tracer kinetic modeling results**

**Plant**	**Age (days)**	**Activity (MBq)**	**Admin. duration (s)**	**ROIs**	**sv (1/s) mean(s.d.)**	**V (mm/s)**	**s = sv/V (1/mm)**
1 RBo019	20	3.18	240	6	0.000287(220)	0.326	0.00088
2 RBo021	20	8.53	300	8	0.000191(058)	0.566	0.00034
3 RBo026	26	9.96	180	5	0.000280(029)	0.214	0.00131
Mean (s.d., n = 3)	22.0(3.5)	7.2(3.6)	240(60)		0.000253(53)	0.368(180)	0.00084(49)

ROIs = number of regions of interest at different positions on stem, sv = trapping probability per unit time; V = velocity up stem, slope of linear regression of position vs mean arrival time; s = Trapping probability per unit length of stem. Corresponding values in units of cm and min, mean(s.d., n = 3): sv = 0.0152(32) 1/min, V = 2.21(1.08) cm/min, s = 0.0084(49) 1/cm.

## Discussion

The temporal resolution and quantitative precision afforded by PET, combined with the petiolar administration of a [^18^F]fluoride bolus into the living plant, permitted kinetic modeling that yielded estimates of ion velocity and trapping fraction in the stem. We observed fluoride transport consistent with bulk flow of water in xylem [18], namely a linear change in position with respect to time. Qualitatively, the time-activity curves that we observed in leaves, reaching a plateau at approximately 10 minutes, agree with a report of [^18^F]fluoride fed to cut stem of soybean [12]. Compared to another report of fluoride transport in the cut stem of soybean [11], the speed we observed in live *Brassica oleracea*, 2.2 cm/min, was 16x slower, and the trapping probability we observed, 0.8%/cm, 1.7x higher. These differences may be attributable to the species studied, environmental conditions in the PET scanner room, physiologic states of the samples, and the presumably lower hydraulic conductance of our plant preparation with intact roots, compared with an open stem.

This study demonstrates the feasibility of imaging basic physiological processes in living plants using a commercially available and widely installed high resolution PET scanner primarily intended for small animal studies. The microPET P4 used in this study easily fits *Brassica oleracea* of up to 4 weeks of age in its 22 cm diameter field of view. The spatial resolution of 2 mm full width at half maximum (FWHM) facilitates delineation of regions of interest to determine time courses in different parts of the plant. Transmission scan capability also permits quantification of the radiotracer temporally and spatially.

In order to model the kinetics of the tracer, we desired a finite duration pulse of fluoride moving through the plant. To that end, we developed a petiolar method of tracer administration. As we reported previously, continuous administration at the roots resulted in constantly increasing uptake in the stem and leaves [14]. With limited administration of a few drops of tracer to the cut petiole, we could observe a finite bolus of activity traveling through the stem permitting application of the kinetic model described here. This petiolar bolus administration was adapted from the petiole-feeding method described by Lin et al. [19]. Because petiole feeding results in the distribution of the fed constituents throughout the entire plant, Lin et al. inferred that translocation was via the phloem and/or apoplast [19]. The images in Figure 1 show whole plant distribution similar to that seen by Lin et al. in their studies using blue dye and radioactive mannitol [19].

The use of relatively widely available scanner technology and a well-established plant model species supports the potential of this method to study plant physiology *in vivo*. Although this study was limited by incomplete quantification because positrons were only partially absorbed [20], the kinetic model that we used was unaffected because it depended only on the shape of the time-activity curves and not the overall scale. As seen in the dynamic image (Figure 1) and the TACs (Figure 2), the tracer appears first below the administration petiole. In an effort to elucidate this behavior, this simple kinetic model of [^18^F]fluoride translocation was also applied downward along the stem below the cut petiole. However, this analysis was unsuccessful (not shown), likely due to the complexity of transport in this region of the stem, which may require multi-compartmental analysis [21].

The accuracy and precision of the measures presented here may be subject to a number of influences. The observed velocity, 2.2 +/− 1.1 cm/min (s.d. n = 3), has a standard error of the mean of +/− 0.6 cm/min, i.e. 95% C.L. 1.0 - 3.4 cm/min, and coefficient of variation of 49%. Some of the variance could be due to differences between the plants and environmental conditions during scanning, while some variance may involve the analysis methods. For instance, all of the free fluoride was assumed to have cleared the regions of interest by the end of the 60 minute scans leaving only an unchanging level of irreversibly trapped fluoride. While this appears to be a plausible assumption in the case of plant RBo019, it may lead to an overestimation of the trapped concentration for the other two plants (Additional file 3: Figure S1). We therefore performed a sensitivity analysis by determining values for sv such that the trapped activity reached only 80% of the final observed activity. This led to relatively small changes in the observed velocities of −2.1% for RBo021 and +3.7% for RBo026, which suggests that 60 minutes is a sufficiently long scan duration. As a further check of the methods, we calculated fluoride arrival times corresponding to the half-height of the free fluoride time activity curves, a simpler technique that uses only the values from the leading edge of the bolus [22]. This yielded comparable velocities (1.6 +/− 1.0 cm/min), albeit with a somewhat higher coefficient of variation, and lends additional confidence to the mean arrival time analysis presented in the Results.

Applications of these techniques currently underway in our laboratory include studies of transport of other substances in living plants. These substances include the ions copper, a crucial micronutrient, chloride, which is important both as a nutrient and a toxin, as well as arsenic, which is an environmental pollutant with significant human health impacts. Understanding the transport behavior of arsenic in plants may aid in advancing methods for bioremediation of arsenic contaminated sites. We are also assessing the mechanisms of uptake and transport of auxins, plant hormones that control growth and other critical processes such as phototropism and gravitropism, using radiotracer imaging by PET. Additionally, physical stress associated with the experimental techniques such as taping to the acrylic plate and bisecting the petiole for radiotracer administration should be further explored and perhaps mitigated [23]. Finally, these techniques may be used to study the influence of environmental conditions such as light, temperature, wind, and humidity on transport processes of various compounds with physiologic relevance to plants.

## Conclusions

The protocol described here utilized a novel combination of techniques to achieve quantitative assessment of fluoride transport in living plants. High resolution commercial small animal PET scanners such as the one used in this work are available for similar studies at many institutions. Petiolar administration permits bolus injection of microliter quantities of radiotracer, and leaves the plant largely intact. This, along with the spatial and temporal resolution of PET allow compartmental kinetic modeling to obtain measures of fluoride velocity and trapping fraction along the stem.

## Methods

### Plants

Experiments were conducted with greenhouse-grown *Brassica oleracea* plants. Seeds of rapid cycling *Brassica oleracea* (RCBC stock #3-012, https://rcbc.wisc.edu) were grown under controlled conditions in 30 cc cell packs filled with a 1:1 mixture of Redi-earth™ and coarse vermiculite (Sun Gro Horticulture Ltd, Canada). Reverse osmosis water was provided through capillary wicking from a reservoir. Each cell/plant received approximately 0.5 g Osmocote™ pellet slow-release fertilizer (14-14-14 NPK) at planting. Additionally, each cell/plant received approximately 25 mL of 2 g/L Peter’s Professional fertilizer (20-20-20 NPK, with micronutrients) at planting and weekly thereafter.

Plants were grown in Madison, WI (43°04’34.1“N 89°25’20.6”W) under a 16 hr photoperiod from May to July in natural light with a daily maximum of 1500 μmol photons m^−2^ s^−1^ photosynthetically active radiation (PAR) supplemented with 500 μmol photons m^−2^ s^−1^ PAR from high pressure sodium lamps. Greenhouse temperature was 21 +/− 3°C. Plants were grown for 20–26 days until they were a suitable size for injection of tracer compounds and scanning.

### [^18^F]Fluoride

[^18^F]Fluoride was produced via the ^18^O(p,n)^18^F nuclear reaction at 16 MeV using the UW-Madison Department of Medical Physics PETtrace cyclotron (GE Healthcare, Uppsala, Sweden) and pure ^18^O-enriched water [24]. This irradiated target water containing [^18^F]fluoride was used directly for plant petiolar administration.

### Scanning procedure and petiolar administration

**Figure 5:**
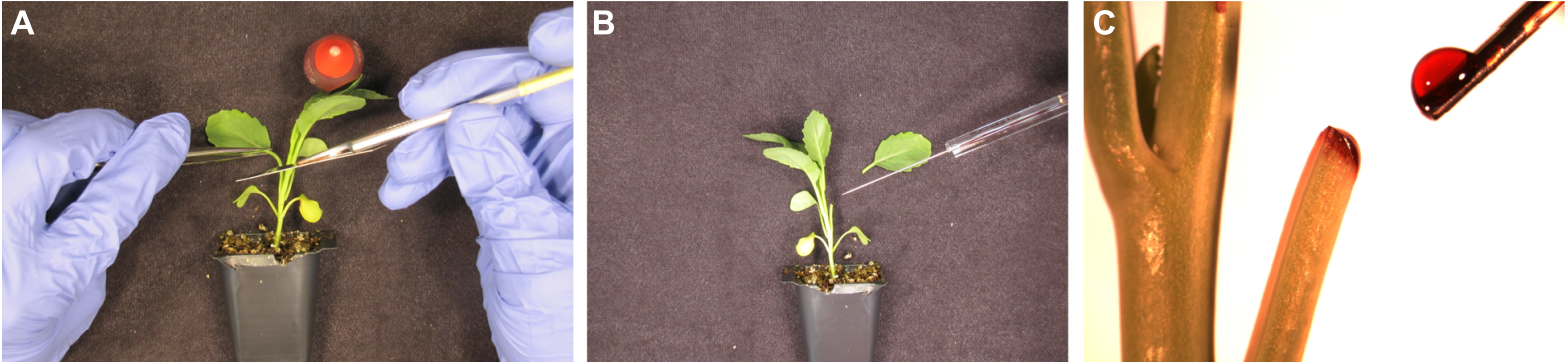
**The petiolar administration method is illustrated here with dye in place of radiotracer and outside the PET scanner for clarity.** The petiole of the first true leaf is transected with a wet scalpel **(A)**, tracer is administered with a liquid chromatography syringe **(B)**, and additional drops are applied before the preceding drop is completely absorbed **(C)**.

Plants were imaged in the UW-Madison microPET P4 scanner (Siemens, Knoxville) with resolution of 2 mm × 2 mm × 2 mm FWHM and 8 cm axial × 19 cm diameter field of view. A plant was placed in a 4.5 cm × 3.0 cm × 4.7 cm hole in a 19.0 cm × 11.5 cm × 5.0 cm block of styrofoam. Directly behind the plant, a slit was cut into the styrofoam such that a 15.2 cm × 11.3 cm × 0.3 cm sheet of acrylic could be mounted behind it. The edges of the plant’s leaves were then taped to the acrylic using paper surgical tape (Micropore™). This resulted in the topside of the leaves being flush against the acrylic with the stomata-rich leaf underside exposed to air. The acrylic was intended to stop positrons and thus increase coincidence counts based on the expectation that over half of [^18^F] positrons escape the parenchyma of a typical leaf [20]. The first true leaf was left untaped to point approximately parallel to the scanner bore, and would be used for the petiolar injection. The plant was then centered in the field of view of the scanner. A ^57^Co transmission scan was acquired (120–125 keV energy window) and then a 60 minute emission scan was begun (350–650 keV, 6 ns coincidence window). Sixty seconds after scan start the petiole was cut using a wet scalpel to ensure that the vasculature remain hydrated and open, and [^18^F] in ^18^O-enriched pure water was applied a drop at a time as each bead was absorbed using a 25 μL liquid chromatography injection syringe (Hamilton) for up to 5 minutes (Figure 5) [19]. No further measures were taken after [^18^F] “injection”. All data were acquired in 3D list mode. Studies were performed at 21 +/− 1°C, typical humidity of 25-45%, and typical light intensity of 200–250 μmol photons m^−2^ s^−1^ PAR.

### Image reconstruction

Image reconstruction was carried out using the scanner vendor’s software (Siemens, microPET Manager 2.4.1.1) For each scan, emission list data were binned into 3D sinograms with time frames of 20×1 + 8×5 minutes (4 byte integers, 168 projection angles x192 bins, span 3 ring difference 31, hist.exe 2.338). For use in attenuation and scatter correction, ^57^Co transmission list data were binned into 3D sinograms, which were then reconstructed by filtered back projection (FBP, full transaxial field of view). Emission images were reconstructed with FBP (fourier 2D rebinning, pixel size 1.90 mm × 1.90 mm in-plane × 1.21 mm slice thickness, ramp filter, recon.exe 2.330). The resulting images included corrections for detector sensitivity, deadtime, decay, attenuation, and scatter.

### Image post processing

The images were visualized using the scanner vendor’s software (Siemens ASIPro VM 6.7.1.2). 2 mm transverse × 2 mm axial Gaussian smoothing was performed on each PET image. The plant petiole, stem, and the junction of the petiole and stem were identified. Circular ROIs of a fixed diameter were drawn using the ASIPro ROI tool. ROI drawing was performed in horizontal planes to cover the cross sectional area of the stem. Depending on the anatomy of the plant, 5 to 8 ROIs were drawn on successive planes beginning at the junction between the petiole and stem and ending when the stem branched into leaves. The ROI closest to the petiole-stem junction was drawn on the first plane where the petiole and stem appeared as two separate circular regions of activity (Additional file 3: Figure S1). Time-activity curves were calculated from the ROIs using the ASIPro software and saved as text files. Before modeling, each time-activity curve was converted to concentration per vertical length of stem per total administered activity.

### Tracer kinetic modeling

A model was implemented based largely on the one described by McKay et al. [11]. Radiotracer is assumed to move unidirectionally up the stem and to be irreversibly trapped at a given position with probability s (1/mm) per unit length. For radiotracer moving at velocity v (mm/s), the trapping probability per unit time is then sv (1/s). Observed radiotracer per unit length at a given position of the stem as a function of time, M(t) (Bq/mm), is assumed to consist of free radiotracer G(t) (Bq/mm) and trapped radiotracer B(t) (Bq/mm), such that

1 $$ \mathrm{M}\left(\mathrm{t}\right)=\mathrm{G}\left(\mathrm{t}\right)+\mathrm{B}\left(\mathrm{t}\right). $$

The rate at which radiotracer is trapped is then given by

2 $$ \mathrm{dB}\left(\mathrm{t}\right)/\mathrm{d}\mathrm{t}=\mathrm{s}\mathrm{v}\ \mathrm{G}\left(\mathrm{t}\right)=\mathrm{s}\mathrm{v}\kern0.5em \left(\mathrm{M}\left(\mathrm{t}\right)\hbox{-} \mathrm{B}\left(\mathrm{t}\right)\right), $$

and integrating over time yields

3 $$ \mathrm{B}\left(\mathrm{t}\right)=\mathrm{s}\mathrm{v}\kern0.5em {\displaystyle \int \left(\mathrm{M}\left(\mathrm{t}'\right)\hbox{-} \mathrm{B}\left(\mathrm{t}'\right)\right)}\kern0.5em \mathrm{d}\mathrm{t}'. $$

Given the initial condition B(t = 0) = 0, the trapped concentration B(t) can then be calculated iteratively for a given set of observed M(t) and the trapping per unit time parameter sv. A value of sv is determined to meet the final condition that at a late time t_f_ all of the observed activity is trapped, i.e. M(t_f_) = B(t_f_). The mid-time of the final frame of PET data, 55 minutes, is taken as t_f_. Once the trapped time-activity curve B(t) has been calculated to meet the final condition, the free time-activity curve is calculated, G(t) = M(t) - B(t) (Additional file 3: Figure S1). Then the mean arrival time of the free radiotracer is given by

4 $$ \mathrm{T}={\displaystyle \int \mathrm{t}\mathrm{G}\left(\mathrm{t}\right)\mathrm{d}\mathrm{t}}/{\displaystyle \int \mathrm{G}\left(\mathrm{t}\right)\mathrm{d}\mathrm{t}}. $$

This mean arrival time was calculated for several positions up the stem and plotted as position vs time (Figure 4). For each plant, the slope of a linear fit to the time-position plot gave the average velocity of the radiotracer traveling up the stem (Additional file 4: Figure S2).

While the integral for Equation 4 would ideally be taken from zero to infinity, the resulting time-position plots were seen to deviate significantly from linearity. This deviation is likely a result of simplifying assumptions in the kinetic model. By limiting the upper bound of the equation 4 integrals, more linear time-position plots were generated, with the upper bound at approximately the half-height of the free time-activity curve for optimal fit. Physically, this corresponds to calculating the mean arrival time of the first 50 to 70% of the free [^18^F]fluoride, likely minimizing the confounding effects of bidirectional fluoride movement and reversible trapping.

The analysis code is available at https://go.wisc.edu/RBoKinetics.

## Additional files

Additional file 1:
**Movie 1.** Uptake over time, 60 1---min frames, 3 mm Gaussian smooth. Additional file 2:
**Movie 2.** Distribution at 40---60 min, 3 mm Gaussian smooth. Additional file 3: Figure S1. Kinetic modeling. left: Regions of interest delineated on PET image of horizontal section of stem. right: Corresponding time-activity curves. blue = observed, red = trapped (model), green = free (model), black = trapped (model) + free (model). Additional file 4: Figure S2. Position vs time plots. Slope of best fit line gives velocity of radiotracer traveling up stem.
