# Supplementary material for: Mathematical modeling of positron emission tomography (PET) data to assess radiofluoride transport in living plants following petiolar administration
Source: Plant Methods. 2015 Mar 13;11:18. doi: 10.1186/s13007-015-0061-y (PMC4359769; doi:10.1186/s13007-015-0061-y)
Supplement: Additional file 3: Figure S1. — Kinetic modeling. left: Regions of interest delineated on PET image of horizontal section of stem. right: Corresponding time-activity curves. blue = observed, red = trapped (model), green = free (model), black = trapped (model) + free (model). [file 13007_2015_61_MOESM3_ESM.pdf]

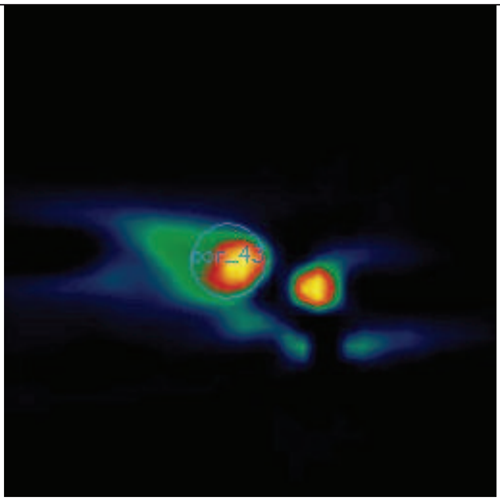

cor_43

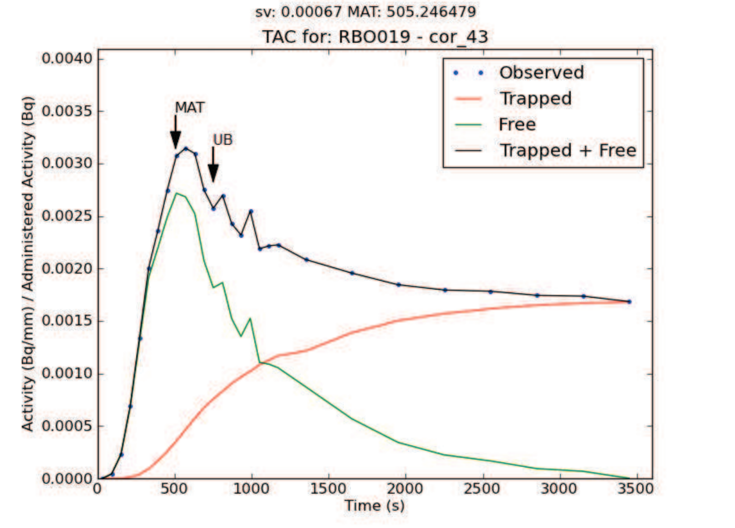

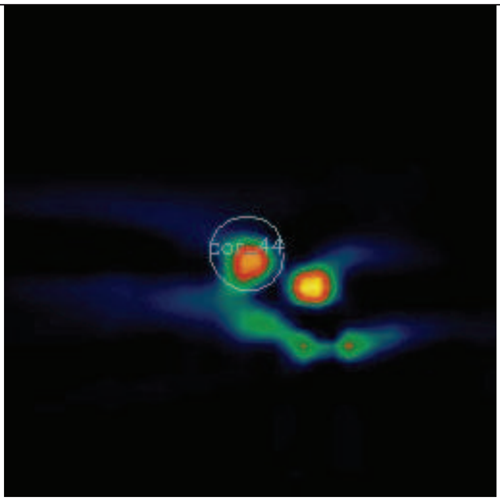

cor_44

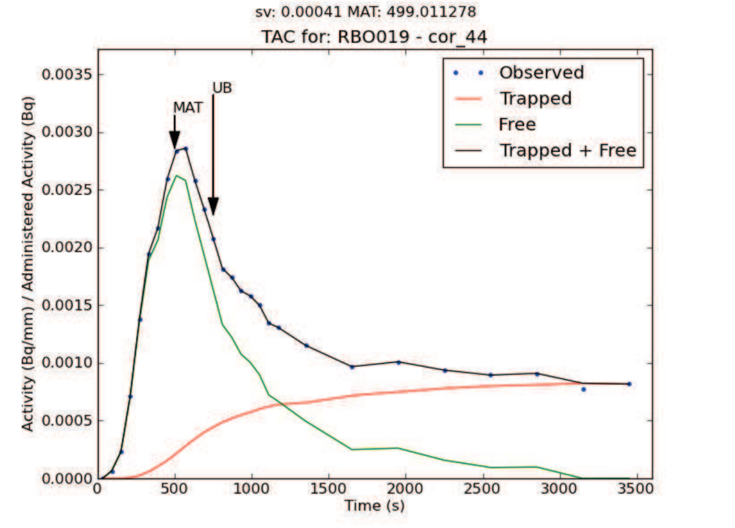

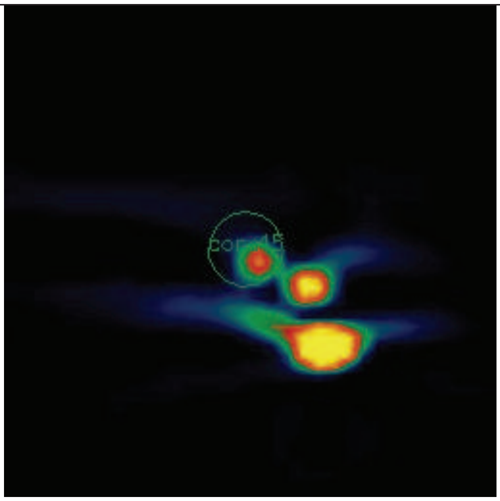

cor_45

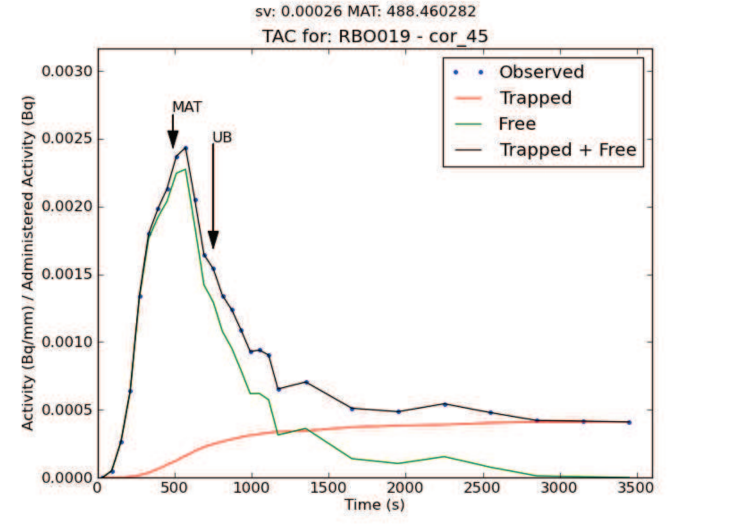

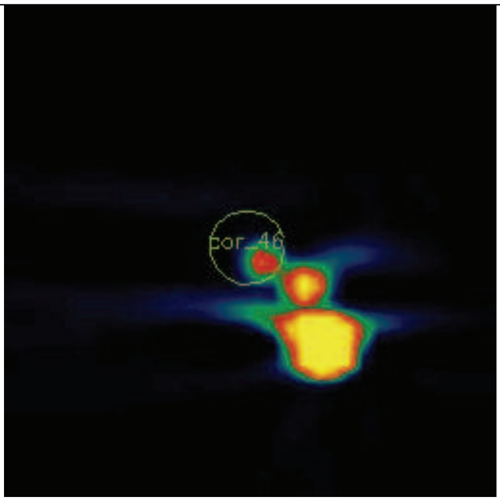

cor_46

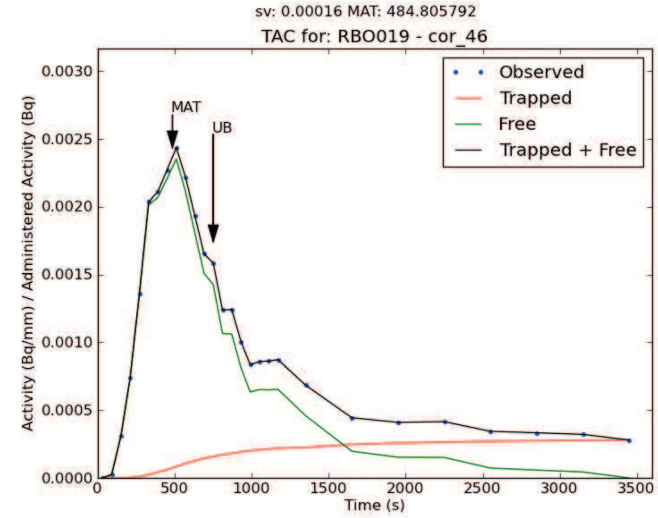

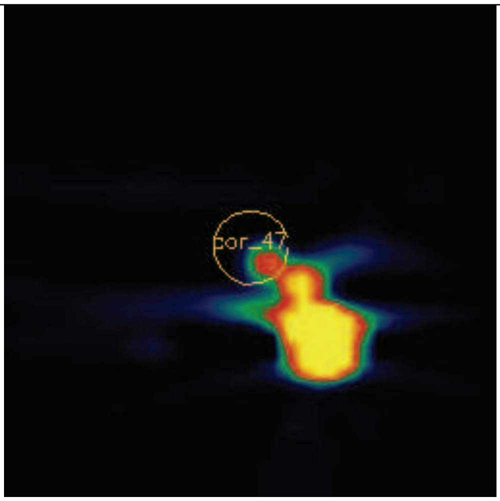

cor_47

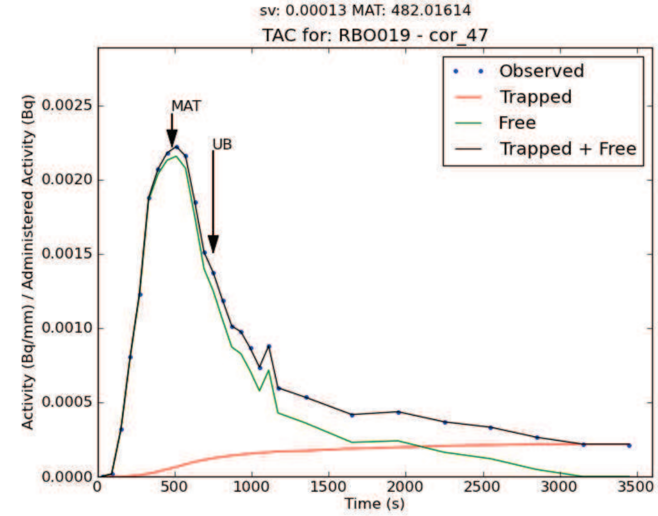

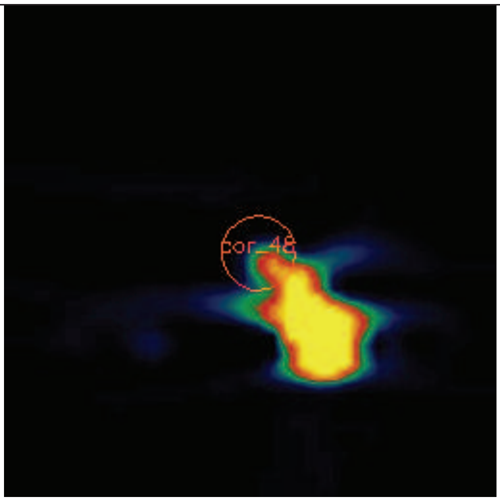

cor_48

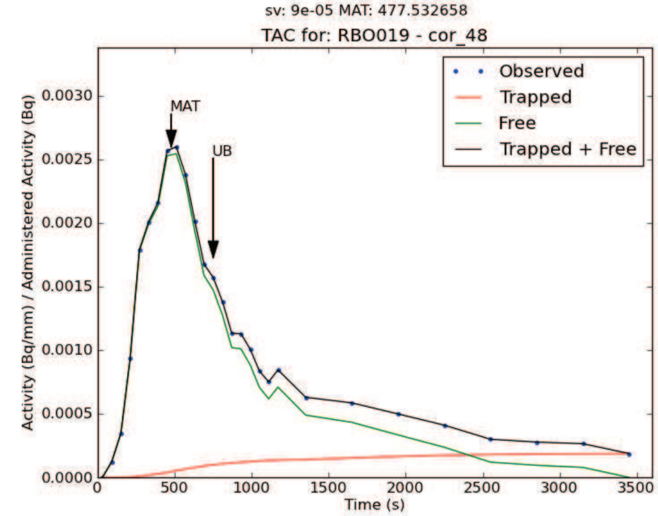

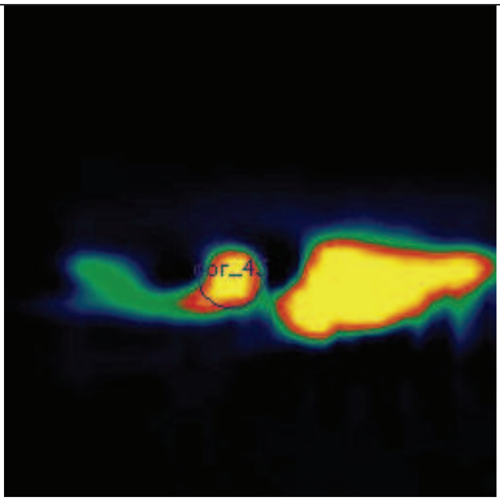

cor_43

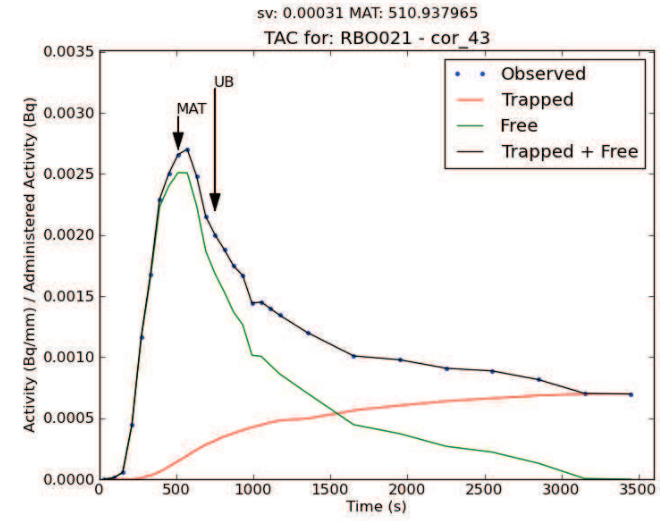

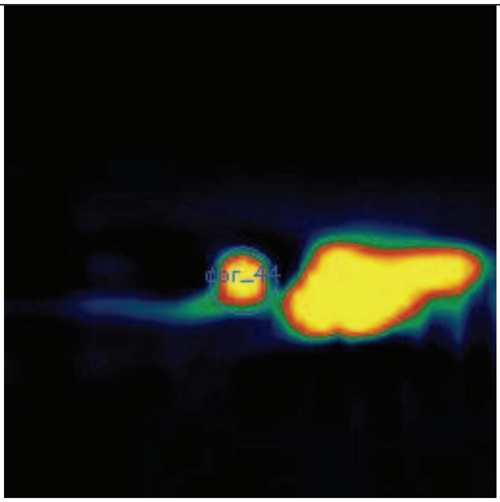

cor_44

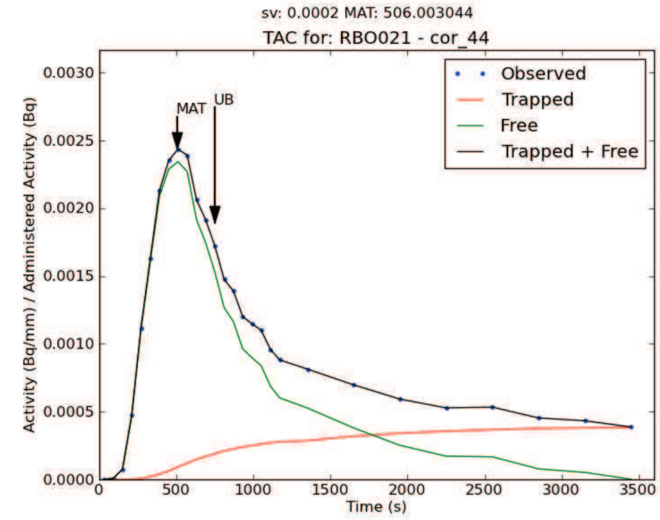

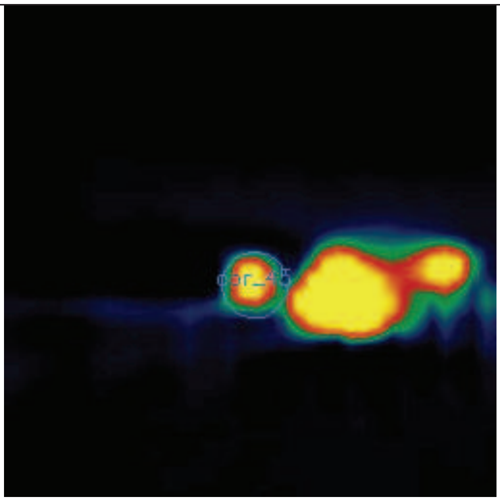

cor_45

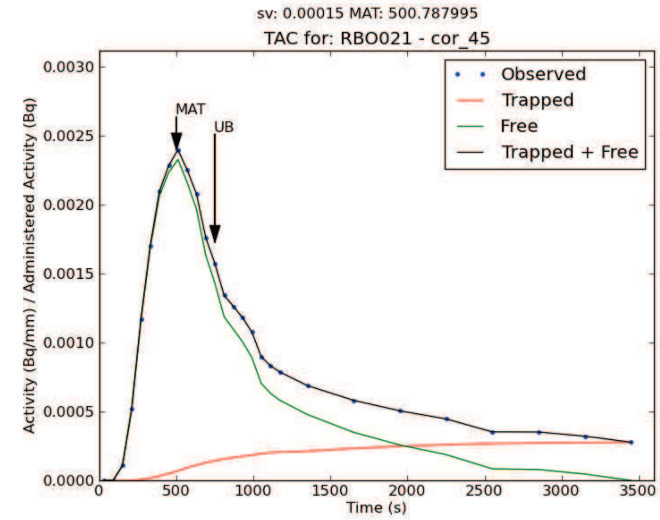

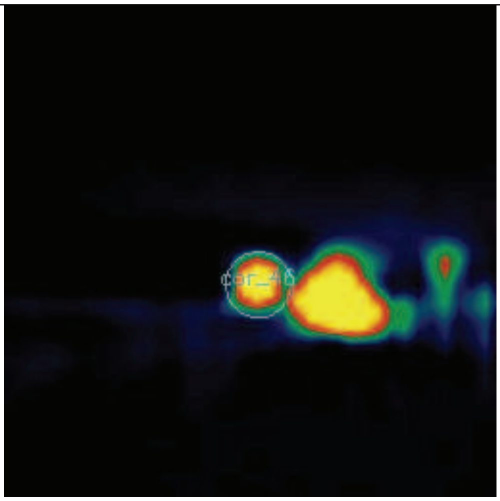

cor_46

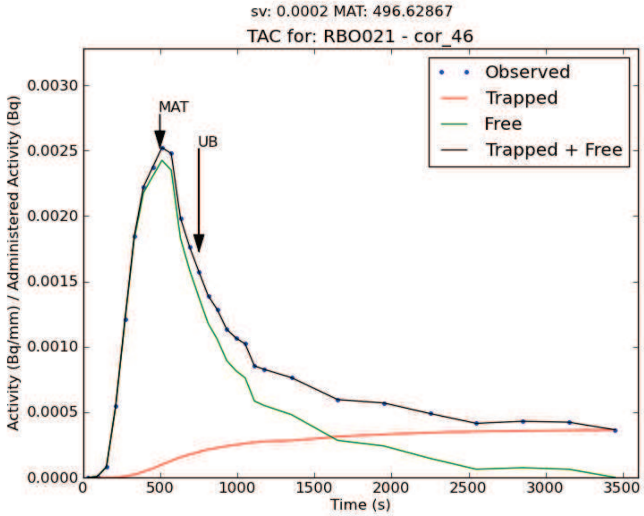

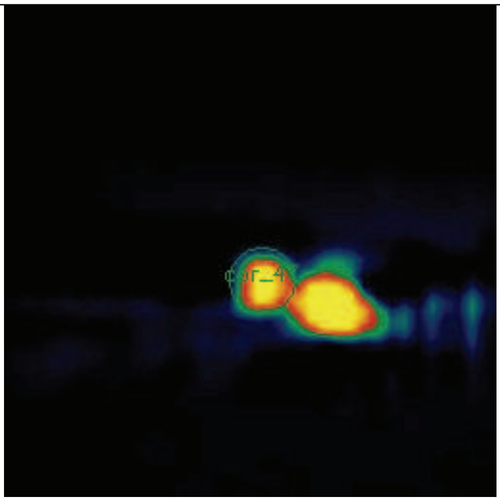

cor_47

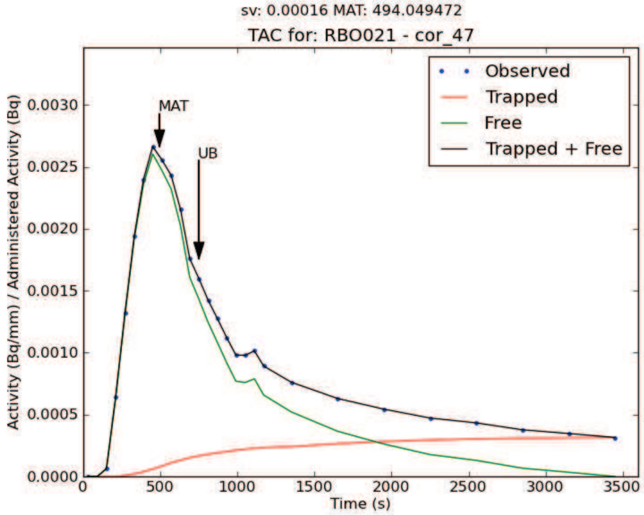

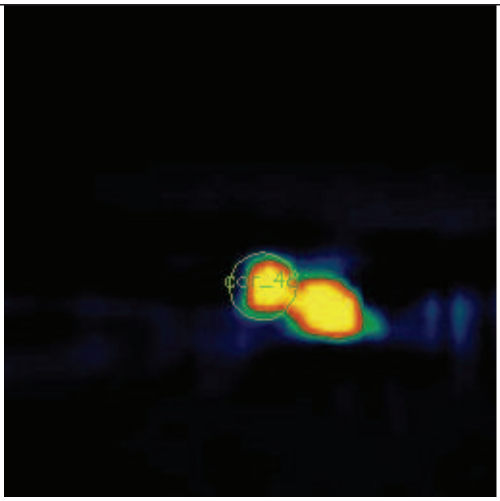

cor_48

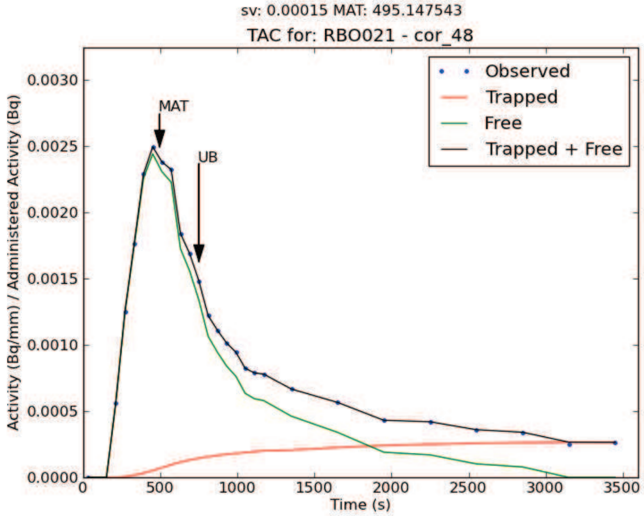

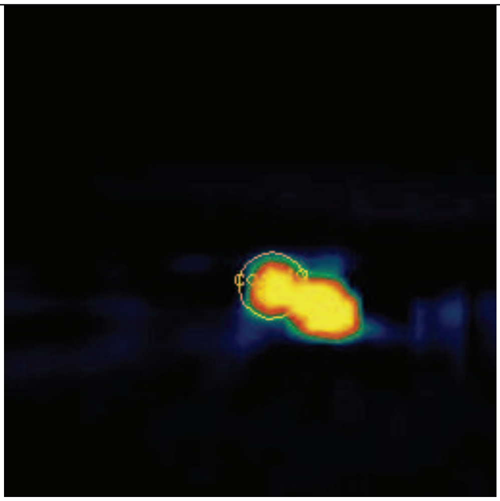

cor_49

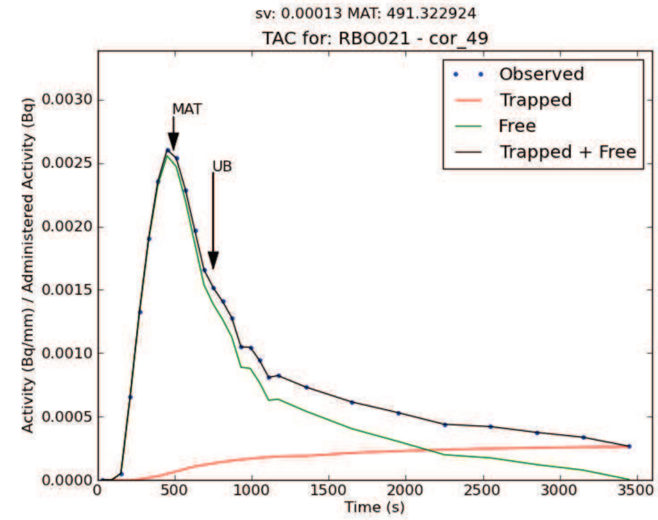

sv: 0.00013 MAT: 491.322924
TAC for: RBO021 - cor_49
Observed
Trapped
Free
Trapped + Free
MAT
UB
Activity (Bq/mm) / Administered Activity (Bq)
0.0030
0.0025
0.0020
0.0015
0.0010
0.0005
0.0000
0
500
1000
1500
2000
2500
3000
3500
Time (s)

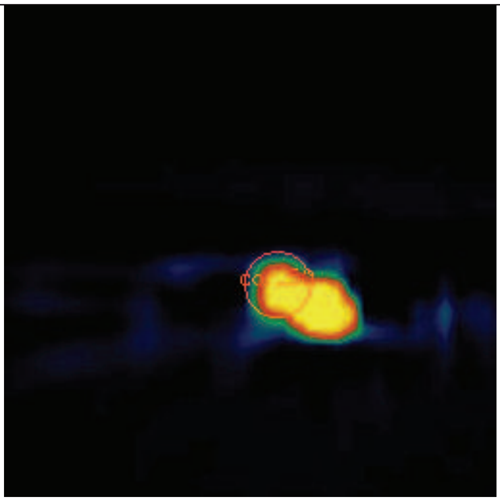

cor_50

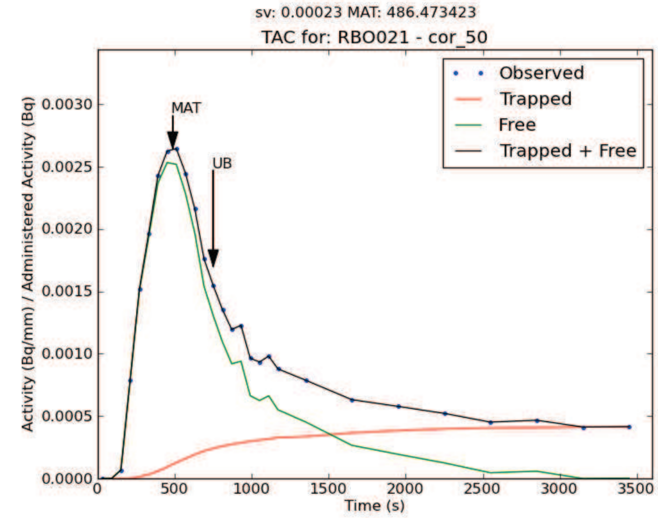

sv: 0.00023 MAT: 486.473423
TAC for: RBO021 - cor_50
Observed
Trapped
Free
Trapped + Free
MAT
UB
Activity (Bq/mm) / Administered Activity (Bq)
0.0030
0.0025
0.0020
0.0015
0.0010
0.0005
0.0000
0
500
1000
1500
2000
2500
3000
3500
Time (s)

RBO026

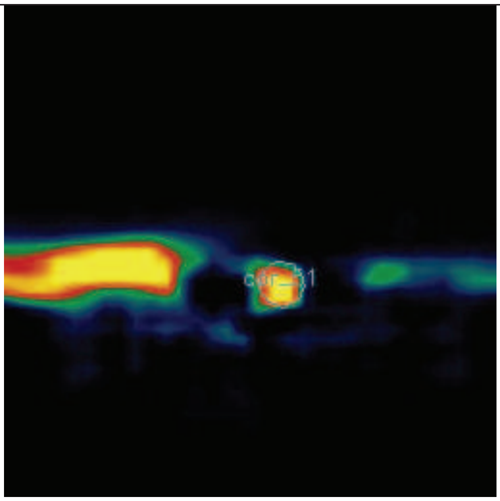

cor_51

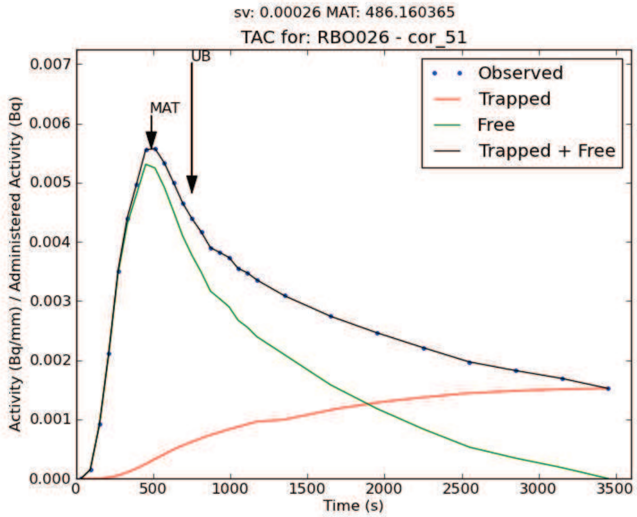

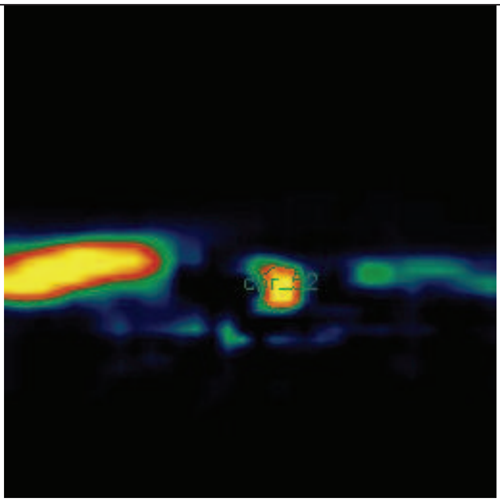

cor_52

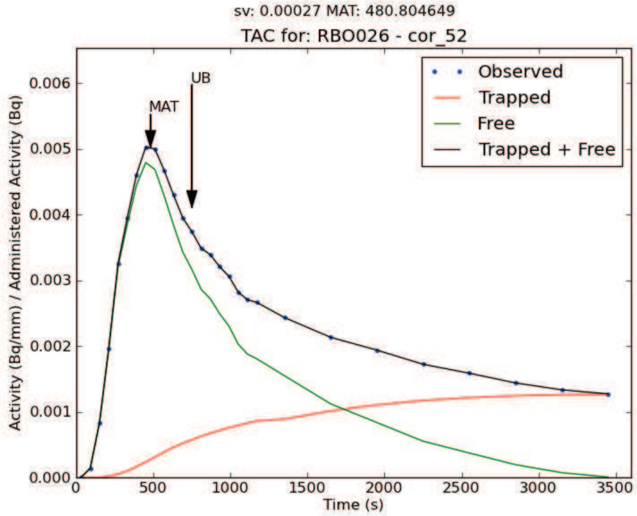

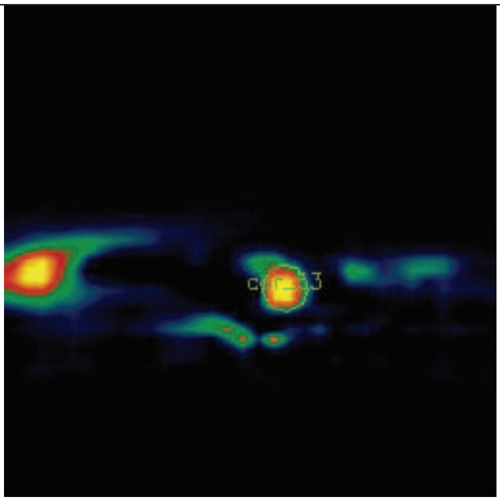

cor_53

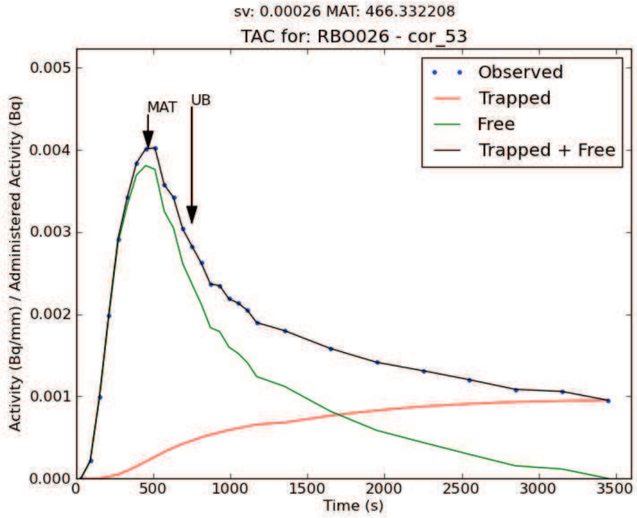

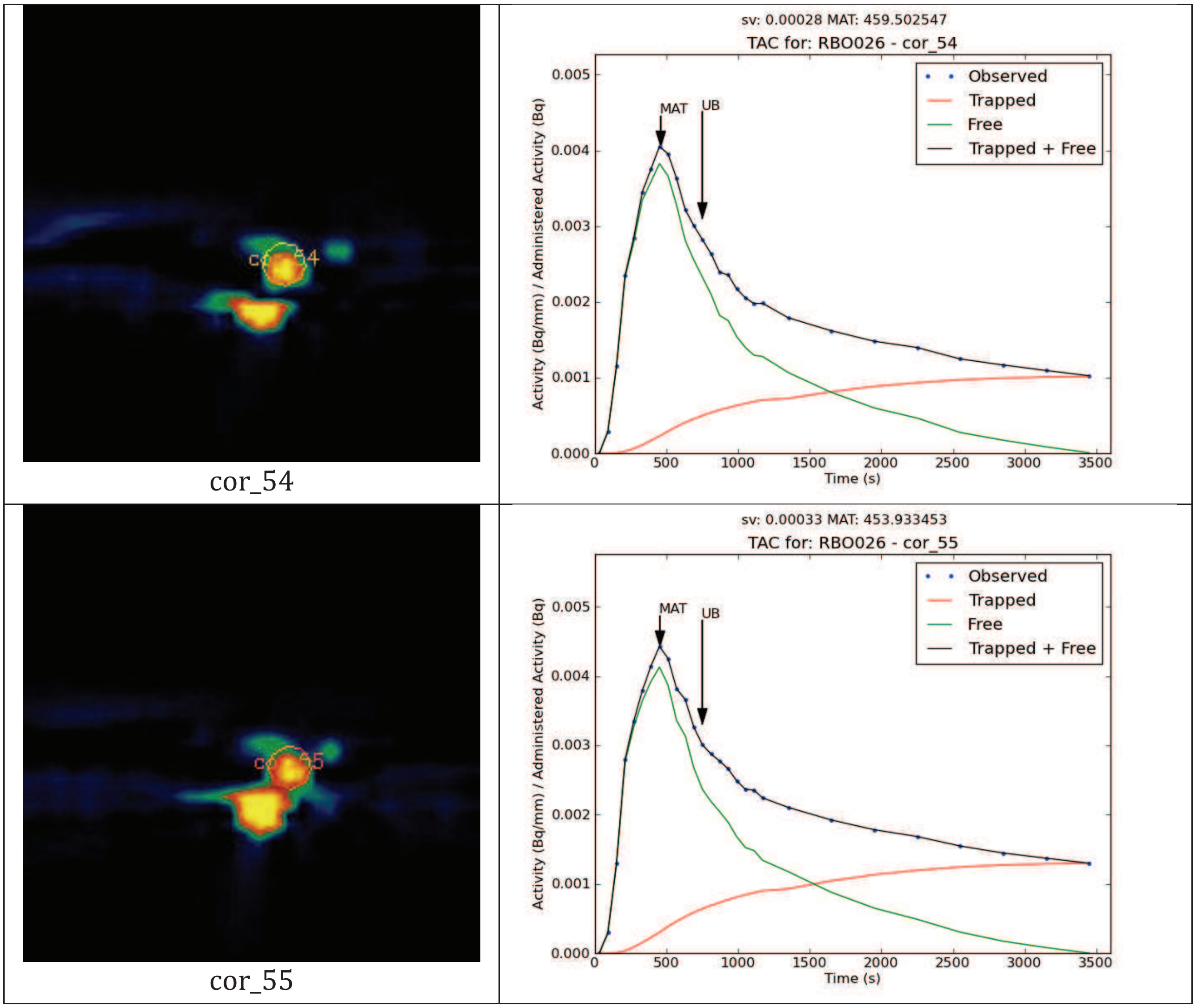

Supplementary Figure 1 Kinetic modeling. left: Regions of interest delineated on PET image of horizontal section of stem. right: Corresponding time-activity curves. blue = observed, red = trapped (model), green = free (model), black = trapped (model) + free (model).
