# Supplementary material for: Mathematical modeling of positron emission tomography (PET) data to assess radiofluoride transport in living plants following petiolar administration
Source: Plant Methods. 2015 Mar 13;11:18. doi: 10.1186/s13007-015-0061-y (PMC4359769; doi:10.1186/s13007-015-0061-y)
Supplement: Additional file 4: Figure S2. — Position vs time plots. Slope of best fit line gives velocity of radiotracer traveling up stem. [file 13007_2015_61_MOESM4_ESM.pdf]

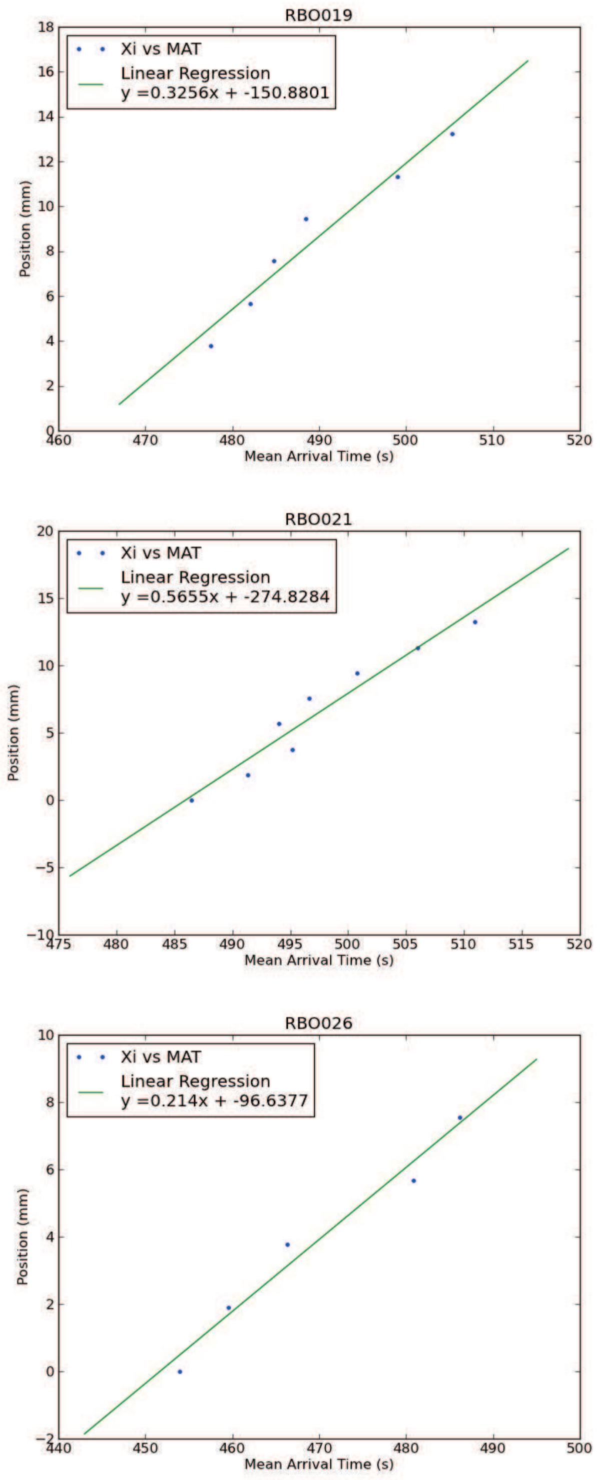

Supplementary Figure 2 Position vs time plots. Slope of best fit line gives velocity of radiotracer traveling up stem.
